# Secular trends in heat related illness and excess sun exposure rates across climatic zones in the United States from 2017 to 2022

**DOI:** 10.1038/s41598-025-93441-3

**Published:** 2025-04-04

**Authors:** Marta Pineda-Moncusí, Rabia Ali Khan, Albert Prats-Uribe, Daniel Prieto-Alhambra, Sara Khalid

**Affiliations:** 1 Centre for Statistics in Medicine (CSM), Nuffield Department of Orthopaedics, Rheumatology and Musculoskeletal Sciences (NDORMS), University of Oxford, Botnar Research Centre, Windmill Road, Oxford, OX3 7LD UK; 2 Epidemiological Insight Team, Advanced Analytics, Analysis and Intelligence Assessment Directorate, Chief Data Officer Group, UK Health Security Agency, London, UK; 3 Department of Medical Informatics, Erasmus University Medical Center, Rotterdam, The Netherlands

**Keywords:** Heat related illness, Heat stress, Excess sun exposure, Climate change, Heatwaves, Electronic health records, GIS, Health inequalities, Observational study, Risk factors, Epidemiology

## Abstract

Heat waves are a major public health challenge, yet the link between heat-related illness (HRI) and regional climate and geography is underexplored. We examined HRI and excess sun exposure incidence rates (IR) [95% confidence interval (CI) per 100,000 person-years], and their correlation with regional maximum temperatures across 9 US climatic zones 33,603,572 individuals were followed from 2017 to 2022. We observed 10,652 individuals with HRI diagnosis (median age: 49 years, 62.3% male). Seasonal peaks occurred during summer: highest overall IR (130.97 [119.93–142.75]) was recorded in July 2019, highest regional IR was reported in the South (186.04 [117.93–279.15]) during 2020. Strongest correlations between monthly maximum temperature and incidence of HRI were observed in the West (Pearson Correlation Coefficient (cor) = 0.854) and Southwest (cor = 0.832). In contrast, we observed 131,204 individuals with excess sun exposure (predominantly older adults [median age: 67 years], 52.3% female, 30% with history of cancer). Overall IR for sun exposure peaked in March 2021 (664.31 [644.84–684.21]) and lacked a consistent seasonal pattern. Sun exposure exhibited weaker correlations with regional temperatures, even in high-temperature regions like the West (cor = 0.305). These data indicate regional variations in HRI. With distinct at-risk groups for HRI and sun exposure, targeted regional interventions may be beneficial, such as heat safety protocols to reduce HRI risk and sun protection campaigns for older adults to mitigate sun exposure risk.

## Introduction

The recent rise in the intensity, duration and frequency of climate-induced heat waves globally is well-documented and projected to further intensify in the coming decades^1–3^. As per the US Environmental Protection Agency, the frequency of heat waves in the US has increased steadily, from an average of two heat waves per year during the 1960s to six per year during the 2010s and 2020s^4^. The rise in heatwaves has significantly increased the incidence of heat-related morbidities resulting in higher hospitalization rates, reduced productivity, and increased premature mortality^1,5–10^.

Heat-related illness (HRI) occurs when the body’s thermoregulatory mechanisms fail to maintain a balance between heat absorption and heat dissipation^11^. This imbalance leads to a dangerous elevation in core body temperature, often triggered by a rise in environmental temperatures or prolonged heat exposure^11,12^. This increase in temperature can lead to a spectrum of health conditions, ranging from mild illnesses such as heat edema, muscle cramps, and heat rash to severe, life-threatening conditions like heat stroke and death^3,11,13,14^. Individuals who recover from exertional heat strokes are at an increased risk of experiencing a recurrence within the first two years following the initial incident, with a relative risk ratio of 3.33 reported amongst participants in the Falmouth Road Race, US^15^.

Despite recovery, some patients may experience lifelong disabilities resulting from neurological and organ damage caused by HRI^16^. HRI can be mitigated through the implementation of effective prevention strategies at the individual, workplace, and community levels. These strategies include acclimatization, maintaining adequate hydration at home and at work, limiting occupational heat exposure during peak summer months and refraining from physically taxing activities in high temperatures, and utilizing residential and community air conditioning places^11,13,17–20^.

In the US, hospitalizations for HRI are most prevalent amongst males, 18–64 years old, particularly those residing in the lowest zip-code income quartile and individuals without health insurance^7,21,22^. Racial and ethnic disparities are evident, with higher HRI hospitalization costs in minority groups such as Blacks, Hispanics, and Asian Pacific Islanders^21^. Certain occupational groups including athletes, military personnel, and outdoor labourers (e.g., farmers, miners, construction workers and firefighters) face an increased risk of developing HRI^11,15,23,24^.

Individuals with obesity, cardiac disease, diabetes mellitus and congenital disorders including ectodermal dysplasia are also at an increased risk of HRI^3,11,25^. Moreover, certain medications including anticoagulants, cardiovascular medicines, nonsteroidal anti-inflammatory drugs (NSAIDs), antipsychotics, antidepressants and anticholinergic agents have been identified as risk factors for HRI due to their impairment of the body’s thermoregulation mechanisms^26,27^. Among these, the concurrent initiation of angiotensin-converting enzyme (ACE) inhibitors and diuretics has been associated with the highest risk of HRI-related hospitalization^26^.

While the incidence of HRI tends to be higher among younger men, it is noteworthy that during the summer of 2020 amid the COVID-19 pandemic in the US, women self-reported a higher incidence of adverse heat effects^28^. Geographically, the same study found that the South and the West of the US reported the highest incidence of self-reported negative heat effects^28^.

Previous studies suggest that as global heat events increase in frequency and severity, and as regional ambient temperatures rise, the associated hospitalization rates, morbidity, and mortality from HRI are also projected to increase, resulting in a greater economic burden and escalating healthcare costs^21,29^.

While the impact and risk factors of HRI have been extensively studied, excess sun exposure remains underexplored as an independent health outcome. Excess sun exposure is a well-established environmental risk factor for periocular cutaneous malignancies and cutaneous melanoma, with research mainly emphasizing its role in melanoma etiology^30–32^. Furthermore, no studies have explicitly separated excess sun exposure cases from HRI cases to determine whether excess sun exposure has a confounding effect on the incidence rate (IR) of HRI.

This study aims to analyse trends in the IR of HRI and sun exposure among individuals across three age groups (0–17 years, 18–64 years, and 65 years and above) in the U.S. from January 2017 to March 2022. Critical evidence gaps persist regarding the impact of COVID-19 on the IR of HRI and the correlation of IR with environmental factors, such as maximum regional temperature. Additionally, no prior studies have characterized patients diagnosed with excess sun exposure to identify at risk groups, geographic hotspots, analysed IR trends within the U.S. population, or investigated how the characteristics of patients with excess sun exposure differ from those with HRI. This study aims to address these gaps, providing novel insights into these two health outcomes.

These data can contribute to the evidence base for public health preparedness and prevention strategies by enabling the effective allocation of resources in healthcare settings to address increase hospitalization rate during the peak seasons and tailoring interventions to reduce HRI and excess sun exposure associated morbidities by identifying at risk groups and regions for each outcome.

## Methods

We conducted an ecological analysis of claims data supplemented with environmental data. The study period includes 01 January 2017 to 31st March 2022.

### Data source

This study utilizes real-world data from the PharMetrics Plus for Academics dataset (IQVIA, USA), a longitudinal adjudicated medical and pharmacy claims database with coverage of more than 150 million commercially insured patients in the US since 2006^33^. The dataset includes patient enrolment data for national and sub-national health plans, and self-insured employer groups. The data is representative of a variety of geographies, employers, payers, providers, and with data from 90% of US hospitals, and over 90% of all US doctors^34^. The dataset used in this study is mapped to the Observational Medical Outcomes Partnership (OMOP) Common Data Model (CDM)^35,36^.

The PharMetrics Plus for Academics dataset was supplemented with regional time-series data on maximum regional temperatures (in degrees Celsius) from 2017 to 2022. This data was sourced from the National Oceanic and Atmospheric Administration’s (NOAA) National Centers for Environmental Information (NCEI)^37^.

The exposure variable includes environmental temperature and sunlight exposure.

### Outcomes

The studied outcomes include HRI and excess sun exposure. HRI included a range of heat related conditions such as heat cramps, heat oedema, heat exhaustion (anhidrotic, due to salt depletion, unspecified), heat fatigue, heat stroke, sunstroke, heat syncope (fainting, light-headedness), heat collapse, contact dermatitis due to hot weather, heat rash, heat stress due to physical exertion, and external injury/accident caused by excessive heat due to weather conditions (Full list of comorbidities, diagnostic codes and concept IDs for HRI are provided in the supplementary information).

The group with the second outcome received a diagnosis for a single comorbidity, specifically exposure to excess sunlight. The corresponding diagnostic code and concept ID are provided in the supplementary information.

The clinical diagnoses were recorded using concept IDs from the (OMOP-CDM) vocabulary. Codes for the outcomes were identified across various vocabularies, including the Systematized Nomenclature of Medicine (SNOMED), Read codes, and the International Classification of Diseases (ICD)-10. These codes were translated into the OMOP vocabulary using their corresponding concept IDs, enabling the standardization of data and ensuring consistency across the different vocabularies used within the OMOP-CDM.

Individuals could contribute multiple events of HRI or excess sun exposure, provided each event was separated by a washout period of at least seven days. This seven-day period was selected based on expert opinion, as it is clinically plausible for a second HRI event to occur at least seven days after the initial event.

### Analysis

Demographic characteristics including sex, age and location of residence, health conditions prior to the index date and medications used within the twelve months preceding the index date were recorded. Median and interquartile range (IQR) were calculated for age. Demographic characteristics such as age groups, health conditions and medications were reported as frequencies and percentages. Health conditions and medications that have been previously identified to predispose individuals to HRI, along with common comorbidities, were included^25,26^. Similar criterion was applied to identify medications included in the analysis, focusing on those potentially associated with the incidence of HRI. The Mantel–Haenszel test was used to calculate p-values for categorical variables, including sex and climatic regions, while a two-sample t-test was applied to compare age distributions between the two groups. Statistical significance was defined using a two-tailed threshold of p < 0.05.

We could not report socio-economic variables such as ethnicity, education level, annual income/occupation and postal code of residence as that information is not available in the OMOP-CDM version of PharMetrics Plus for Academics.

Overall monthly incidence rates (IR) calculated as number of cases per 100,000 person-years (95% confidence interval) for HRI and excess sun exposure were calculated and stratified by sex and age groups (< 18, 18–64 and ≥ 65 years), and by NCEI’s nine climatically consistent regions i.e. Northwest, West, Northern Rockies and Plains, Southwest, Upper Midwest, South, Ohio Valley, Southeast and Northeast^38^. These regions were originally identified by Karl et al. in their 1984 study through climate analysis and this classification is currently being used by the NCEI for grouping data into regions^38,39^. Given that the PharMetrics® Plus for Academics dataset includes individuals from Alaska, American Samoa, Guam, Hawaii, the Northern Mariana Islands, Puerto Rico, the Trust Territories, and the U.S. Virgin Islands, areas not classified within the NCEI’s nine climatic regions, these locations were grouped into an “Other” category for analysis.

Monthly IRs were computed from January 2017 to March 2022, with data censored beyond March 2022. The onset of the COVID-19 lockdown restrictions in the US, which began on March 15, 2020, is marked by a red dashed line on the plots to clearly indicate this temporal reference point.

The correlation between maximum regional temperature and the IR of HRI and excess sun exposure were assessed for each climatic region using the Pearson Correlation Coefficient method. Coefficient values between ± 0.50 and ± 1 were classified as strong correlations, values between ± 0.30 and ± 0.49 were considered moderate correlations, and values below ± 0.29 were categorized as weak correlations.

All analysis was conducted using RStudio Server (2023.03.0 + 386 “Cherry Blossom” Release) driven by R Version 4.2.3. 

The PharMetrics Plus for Academics dataset did not require an internal review board (IRB) approval for use, as it comprises pseudo-anonymized secondary data, ensuring compliance with patient privacy and confidentiality standards.

## Results

A total of 141,856 individuals were identified as having either HRI (n = 10,652) or excess sun exposure (n = 131,204) from the larger study cohort of 33,603,572 individuals between January 2017 to March 2022 (Table 1). A total of 181 individuals overlapped between the two groups.

**Table 1:** Demographic Characteristic of Patients with Heat Related Illness and Excess Sun Exposure at the Time of First Diagnosis*.

	Heat related illness	Excess sun exposure	p-value
Overall number of patients	10,652	131,204	
Women (n (%))	4,019 (37.7%)	67,282 (51.3%)	< 0.001
Men (n (%))	6,633 (62.3%)	63,922 (48.7%)	
Median age (years (IQR))	49 (28–66)	67 (57–77)	< 0.001
Overall number of outcomes	12,998	272,604	
Number of patients by age group (n)			
0 to 17	1,158 (10.87%)	1,096 (0.84%)	< 0.001
18 to 64	6,685 (62.76%)	54,724 (64.59%)	
> 65	2,809 (42.02%)	75,384 (57.46%)	
Number of outcomes by age group (n)			
0 to 17	1,213 (9.33%)	1,324 (0.49%)	
18 to 64	7,383 (56.80%)	84,736 (31.08%)	
> 65	4,402 (33.87%)	186,544 (68.43%)	
Location of patient’s residence (US Climate Regions):			
Southeast	2,065 (19.4%)	17,383 (13.2%)	< 0.001
Southwest	494 (4.6%)	14,672 (11.2%)	
Northwest	311 (2.9%)	5,886 (4.5%)	
West	1,372 (12.9%)	30,919 (23.6%)	
Northeast	1,428 (13.4%)	18,814 (14.3%)	
South	928 (8.7%)	4,027 (3.1%)	
Ohio Valley	3,105 (29.1%)	32,260 (24.6%)	
Upper Midwest	840 (7.9%)	5,571 (4.2%)	
North Rockies and Plains	37 (0.3%)	298 (0.2%)	
Other**	9 (0.1%)	162 (0.1%)	
NA***	63 (0.6%)	1,212 (0.9%)	

*IQR* Interquartile Range, *US* United States of America.

*A total of 181 individuals overlapped between the two outcome groups.

**Patients from Alaska, American Samoa, Guam, Hawaii, Northern Mariana Islands, Puerto Rico, trust Territories and Virgin Islands are included in the ‘Other’ region.

***Individuals categorised in NA have no region identified in their records.

The HRI cases reported a median age of 49 (IQR: 28–66) years, consisted predominantly males (n = 6,633, 62.3%) and approximately a third belonged to the Ohio Valley (n = 3,105, 29.1%) followed by the Southeast (n = 2,065, 19.4%). Hypertension (n = 4,143, 39.0%), anxiety (n = 1992, 18.7%), gastroesophageal reflux disease (n = 1,683, 15.8%), depressive disorder (n = 1,536, 14.4%) and diabetes (n = 1,484, 13.9%) were the top 5 commonly reported comorbidities (Table 2).

**Table 2:** Comorbidities and Medication Use Among Patients with Heat Related Illness and Excess Sun Exposure at the Time of First Diagnosis.

Conditions any time prior to diagnosis	Heat related illness	Excess sun exposure
Chronic Obstructive Pulmonary Disease	594 (5.6%)	7973 (6.1%)
Inflammatory bowel disease	85 (0.8%)	1521 (1.2%)
Anxiety	1992 (18.7%)	21,461 (16.4%)
Stroke	251 (2.4%)	2994 (2.3%)
Asthma	902 (8.5%)	10,519 (8.0%)
Gastroesophageal reflux disease	1683 (15.8%)	26,979 (20.6%)
Malignant neoplastic disease	904 (8.5%)	38,755 (29.5%)
Dementia	265 (2.5%)	2635 (2.0%)
Hypothyroidism	974 (9.1%)	20,091 (15.3%)
Chronic liver disease	98 (0.9%)	895 (0.7%)
HIV	23 (0.2%)	191 (0.1%)
Heart failure	510 (4.8%)	7282 (5.6%)
Rheumatoid arthritis	107 (1.0%)	2645 (2.0%)
Autoimmune disease	634 (6.0%)	12,862 (9.8%)
Pneumonia	467 (4.4%)	6183 (4.7%)
Osteoporosis	329 (3.1%)	9995 (7.6%)
Venous thromboembolism	163 (1.5%)	2837 (2.2%)
Hypertension	4143 (38.9%)	62,679 (47.8%)
Diabetes	1484 (13.9%)	17,419 (13.3%)
Depressive disorder	1536 (14.4%)	15,657 (11.9%)
Chronic kidney disease	788 (7.4%)	13,219 (10.1%)
Myocardial infarction	183 (1.7%)	1882 (1.4%)
Medications reported in the twelve months preceding the diagnosis		
Antineoplastic agents	228 (2.1%)	6207 (4.7%)
Immunosuppressants	118 (1.1%)	2539 (1.9%)
Drugs used in diabetes	911 (8.6%)	7609 (5.8%)
Drugs acid related disorder	1356 (12.7%)	13,487 (10.3%)
Drugs for obstructive airway diseases	1399 (13.1%)	14,250 (10.9%)
Agents acting on the renin-angiotensin system	2042 (19.2%)	22,543 (17.2%)
Diuretics	876 (8.2%)	11,531 (8.8%)
Hormonal contraceptives for systemic use	389 (3.7%)	3142 (2.4%)
Lipid modifying agents	1876 (17.6%)	27,144 (20.7%)
Antibacterials for systemic use	3471 (32.6%)	35,595 (27.1%)
Antiepileptics	1039 (9.8%)	8141 (6.2%)
Calcium channel blockers	873 (8.2%)	10,458 (8.0%)
Antidepressants	1689 (15.9%)	15,525 (11.8%)
Antinflammatory antirheumatic	3146 (29.5%)	35,331 (26.9%)
Psycholeptics	1548 (14.5%)	15,565 (11.9%)
Antithrombotics	788 (7.4%)	8482 (6.5%)
Opioids	1382 (13.0%)	13,457 (10.3%)
Psychostimulants	284 (2.7%)	1350 (1.0%)
Beta blocking agents	1127 (10.6%)	14,650 (11.2%)

A total of 181 individuals overlapped between the two outcome groups.

In contrast, the excess sun exposure cases were older, with a median age of 67 (IQR: 57–77) years. The group reported a nearly even gender distribution (n = 67,282, 51.3% females) and a higher proportion resided in Ohio Valley (n = 32,260, 24.6%), followed by the West (n = 30,919, 23.6%) (p < 0.001). The top 5 most prevalent comorbidities were identified as hypertension (n = 62,679, 47.8%), malignant neoplastic diseases (n = 38,755, 29.6%), gastroesophageal reflux disease (n = 26,979, 20.6%) and anxiety (n = 21,461,16.4%).

It is noteworthy that individuals aged 18 years and under comprise 10% (n = 1,158) of HRI cases but only 0.84% (n = 1,096) of excess sun exposure cases (p < 0.001).

Antibacterials and anti-inflammatory medications were the most commonly used medications in both groups, with comparable prevalence observed in the HRI cases compared to the excess sun exposure cases. In the HRI cases, 32.6% (n = 3,471) of individuals used antibacterials and 29.5% (n = 3,146) used anti-inflammatory medications, compared to 27.1% (n = 35,595) and 26.9% (n = 35,331), respectively, in the excess sun exposure cases.

## Heat related illness

### Overall monthly incidence rate of heat related illness from January 2017 to March 2022

**Fig. 1:**
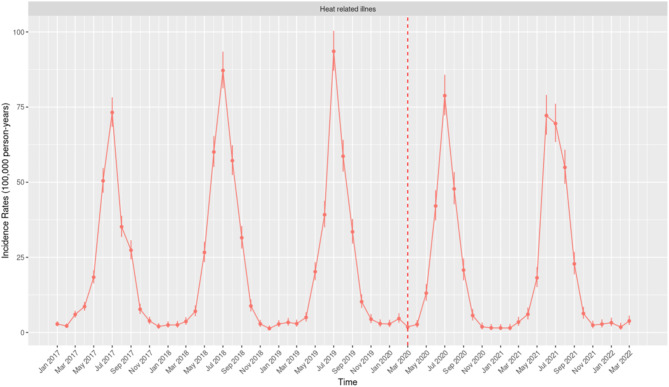
Monthly Incidence Rates of Heat Related Illness per 100,000 person-years in the United States of America from January 2017 to March 2022.

The IR of HRI exhibits pronounced seasonal patterns, with consistent annual peaks during the summer months (June–August) with the highest rates typically observed in July, except in 2021 when the peak occurred in June (Fig. 1). The highest IR was recorded in July 2019 at 130.97 per 100,000 person-years (95% CI: 119.93–142.75), followed by July 2018 with an IR of 114.39 (95% CI: 104.59–124.86). A significant reduction in the IR of HRI was noted during the winter months, with the lowest IR in March 2020 at 1.18 (95% CI: 0.39–2.76).

Although the seasonal pattern for IR remained consistent, a notable decline in peak IR values was observed during the two summers following March 2020. In 2020, the peak IR occurred in July, declining to 78.80 (95% CI: 72.28–85.76), while in 2021, the peak shifted to June and further declined to 72.18 per 100,000 person-years (95% CI: 65.78–79.04).

### Monthly incidence rate of heat related illness in the United States stratified by sex and age groups

**Fig. 2:**
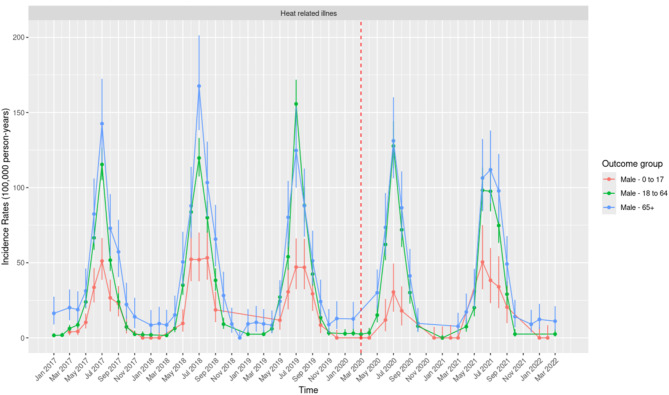
Monthly Incidence Rates of Heat Related Illness per 100,000 person-years in the United States of America from 1 January 2017 to March 2022, for Males Across the Three Age Groups.

Males aged 18–64 years, and those aged 65 years and older consistently exhibited the highest monthly IRs of HRI (Fig. 2). Among males aged 65 years and older, the highest annual IRs were recorded in July 2018 (167.62; 95% CI: 138.27–201.37), followed by July 2017 (142.59; 95% CI: 116.74–172.46) and July 2020 (131.15; 95% CI: 106.23–160.16).

In 2019, males aged 18–64 years reported the highest annual IR (155.74; 95% CI: 140.79–171.84). Notably, in 2020, the IRs for the two groups were nearly equal, with males aged 18–64 years reporting an IR of 127.69 (95% CI: 112.67–144.16) and those aged 65 years and older an IR of 131.15 (95% CI: 106.23–160.16).

Seasonality in the IR of HRI is evident across males and all age groups, with annual peaks consistently observed during the summer months (June–August). The impact of COVID-19 restrictions on the IR of HRI varied between males aged 65 years and above, and those aged 18–64 years. Among males aged 65 and above, the IR in summer 2020 was higher than the previous year, peaking at levels comparable to July 2019, with slight declines observed in July 2021. Conversely, males aged 18–64 years experienced a more substantial reduction in IR during the summer of 2020, which was further reduced in the summer of 2021, reflecting a more pronounced downward trend in this age group.

**Fig. 3:**
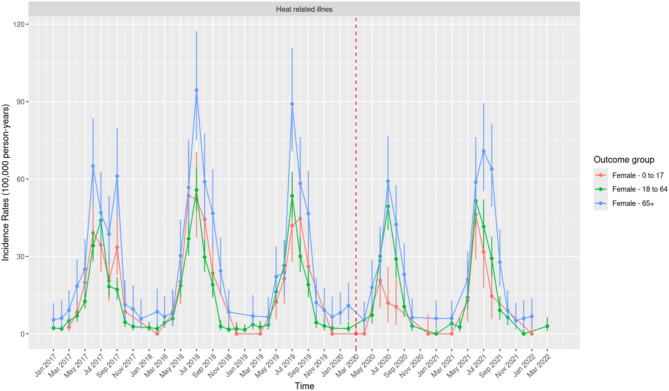
Monthly Incidence Rates of Heat Related Illness per 100,000 person-years in the United States of America from 1 January 2017 to March 2022, for Females Across the Three Age Groups.

Seasonality in the IR of HRI is evident across females of all age groups, with annual peaks consistently observed during the summer months (June–August) (Fig. 3). Among females, those aged 65 years and above exhibited the highest IRs of HRI, particularly during the summer months. Highest IR in this demographic group was recorded in July 2018 (94.47; 95% CI: 75.14–117.27), followed by July 2019 (89.10; 95% CI: 70.76–110.75). Females aged 18–64 years demonstrated moderate IRs, while those aged 0–17 years consistently reported the lowest IRs, highlighting significant age-specific disparities in HRI risk.

Females aged 65 years and above experienced a marked decline in IR during the summer of 2020, significantly lower than pre-pandemic levels. In contrast, females aged 18–64 years showed stable IRs across 2020 and 2021, with no significant changes compared to pre-pandemic years.

### Monthly incidence rate of heat related illness in the US stratified by climate regions

**Fig. 4:**
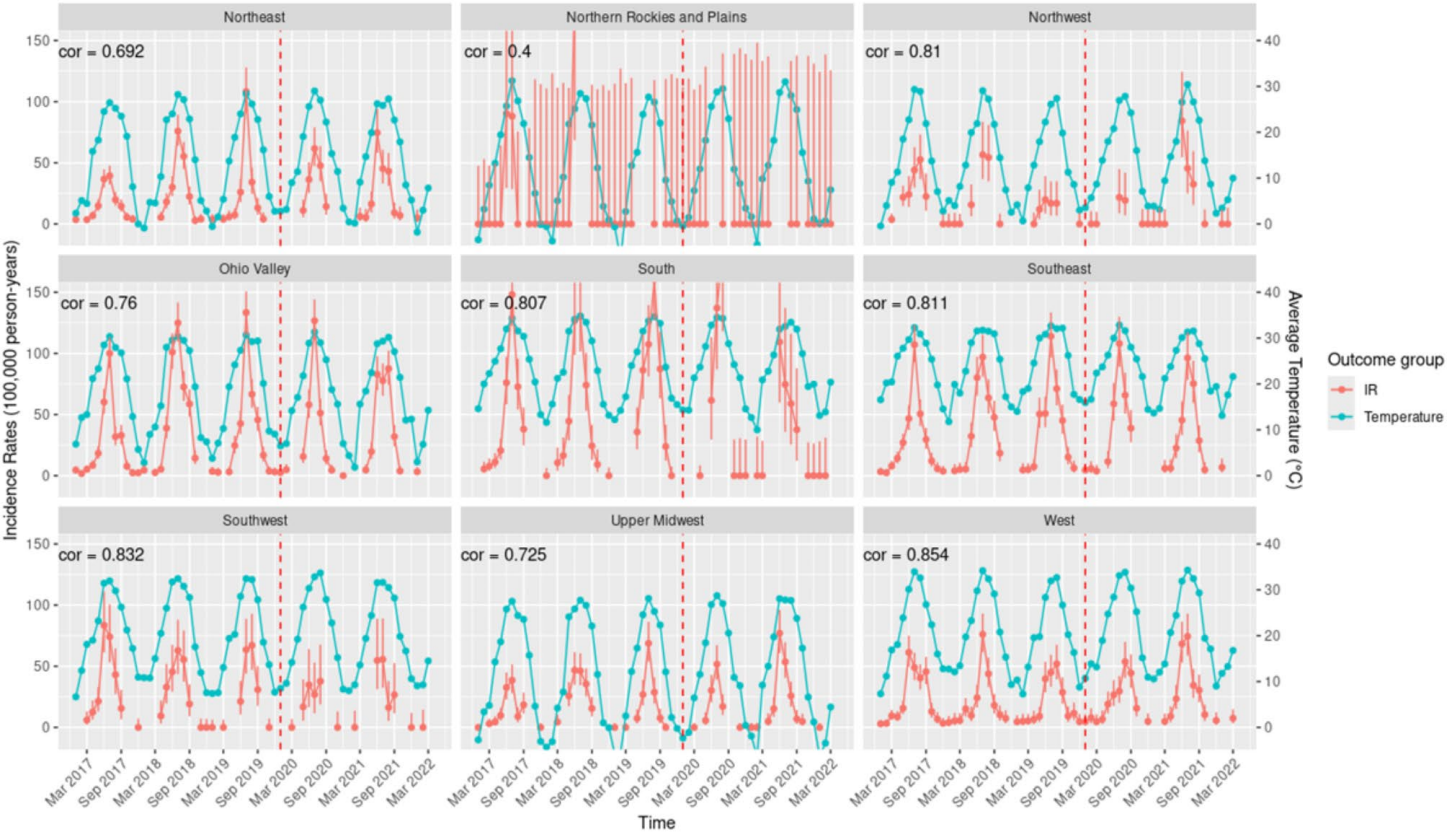
Monthly Incidence Rate of Heat Related Illness in the Nine Climate Regions of the United States of America from January 2017 to March 2022.

IR for HRI demonstrated consistent seasonal trends across all regions of the US, with peaks in the summer and lowest rates during the winter months (Fig. 4). Although the Northern Rockies and Plains reported the highest monthly IR in June 2018 (186.60; 95% CI: 68.48–406.15), the small sample size (n = 35) and limited number of events (n = 6) limits the generalizability of this finding. Therefore, we consider the values reported for the South region, which had a larger and more representative sample size and number of events, to be more accurate and reflective of population-level trends. Hence, the highest IR of HRI across all regions was reported in the South region in August 2020, with an IR of 186.04 [95% CI: 117.93–279.15]. The second-highest IR was also observed in the South region, occurring in August 2019, with an IR of 160.02 [95% CI: 127.45–198.37].

Strong positive correlations between changes in maximum regional temperature and IR of HRI were observed in the West (cor = 0.854), Southwest (cor = 0.832) and Southeast (cor = 0.811), South (cor = 0.807), Northwest (cor = 0.81), and Ohio Valley (cor = 0.76) regions, where IR peaks aligned closely with high temperatures, indicating a strong temperature-dependent pattern. The Northern Rockies and Plains reported the weakest correlation (cor = 0.4). The COVID-19 pandemic did not alter the seasonal relationship between maximum regional temperature and IR for HRI except for in Northeast and the Southwest.

## Excess sun exposure

### Overall monthly incidence rate of excess sun exposure from January 2017 to March 2022

**Fig. 5:**
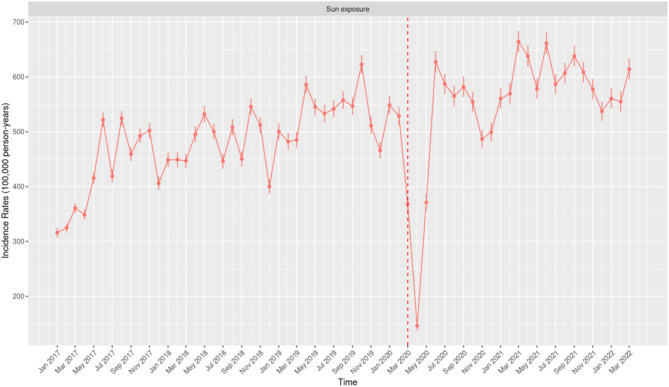
Monthly Incidence Rates of Excess Sun Exposure per 100,000 person-years in the United States of America from January 2017 to March 2022.

The IR of excess sun exposure in the overall group exhibited a steady increasing trend from 2017, reaching a peak in March 2021 (664.3; 95% CI: 644.84–684.21), followed by the second-highest IR in June 2021 (661.35; 95% CI: 641.66–681.49) (Fig. 5). A significant decline in IR was observed following the onset of the COVID-19 restriction, with the lowest IR reported in April 2020 at 146.03 (95% CI: 137.68–154.77). This was followed by a sharp rebound in the subsequent months, with IRs exceeding pre-pandemic levels.

### Incidence rate of excess sun exposure stratified by sex and age from January 2017 to March 2022

**Fig. 6:**
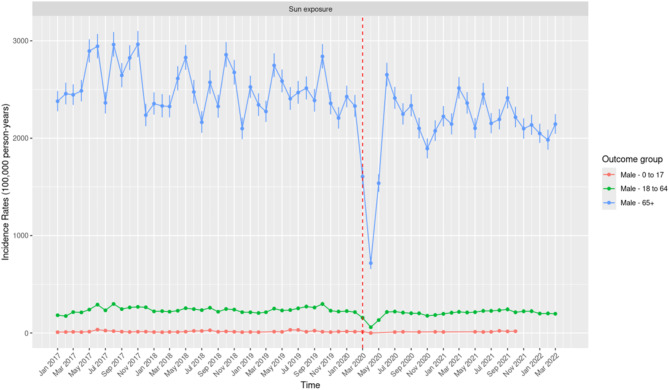
Monthly Incidence Rates of Excess Sun Exposure Among Males Stratified by Age Groups (0–17 Years, 18–64 years, and 65 + Years) in the United States of America from January 2017 to March 2022.

**Fig. 7:**
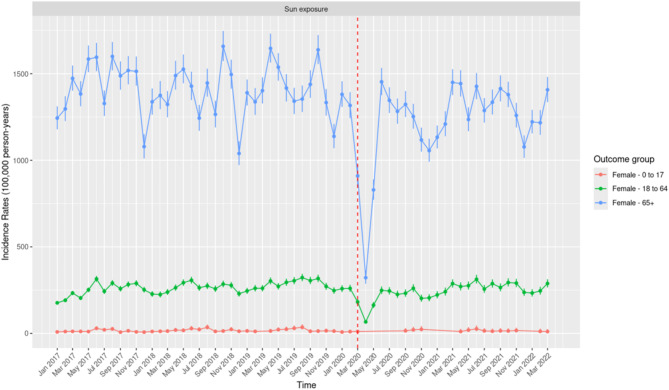
Monthly Incidence Rates of Excess Sun Exposure Among Females Stratified by Age Groups (0–17 Years, 18–64 Years, and 65 + Years) in the United States of America from January 2017 to March 2022.

Males aged 65 years and above consistently reported the highest IR compared to all other demographic groups, including males aged 0–17 and 18–64 years, as well as females across all age groups (Fig. 6 and Fig. 7). The highest IR for males aged 65 years and above was recorded in June 2017 at 2,964.00 (95% CI: 2,832.17–3,100.37), with a secondary peak observed in August 2020 at 2,961.67 (95% CI: 2,836.63–3,090.80). A sharp decline in IR occurred in April 2020 (717.28 per 100,000 person-years; 95% CI: 657.28–781.30) following the onset of COVID-19 restrictions. This decline was followed by a significant rebound in June 2020, with an IR of 2,651.00 (95% CI: 2,533.24–2,774.75). Males aged 0–17 years and 18–64 years consistently exhibited low IRs for excess sun exposure.

Similar to males, females aged 65 years and above consistently reported the highest IRs of excess sun exposure compared to the two younger age groups, although these IRs were consistently lower than those observed in males aged 65 years and above (Fig. 7). The highest IR was observed in November 2018 at 1,658.18 (95% CI: 1,573.26–1,746.50), followed by April 2019 with an IR of 1,646.23 (95% CI: 1,564.29–1,731.35). The lowest IR occurred in April 2020 at 322.01 (95% CI: 286.77–360.38) following the COVID-19 restrictions.

In contrast to the seasonality reported in IR of HRI, the IRs of excess sun exposure for males and females aged 65 years and above do not consistently align with specific seasons, such as summer or winter. Peaks were observed in months like October/November and April, which fall outside traditional "high sun exposure" periods. While fluctuations in IRs were noted for this demographic, the variation does not follow a clear seasonal pattern across years.

### Incidence rate of excess sun exposure stratified by the nine climate regions in the US from January 2017 to March 2022

The Southwest consistently reported the highest IRs for excess sun exposure among all regions. The peak IR occurred in March 2021 at 1549.27 (95% CI: 1411.49–1696.87), followed by another high IR in June 2020 at 1484.03 (95% CI: 1346.59–1631.69).

**Fig. 8:**
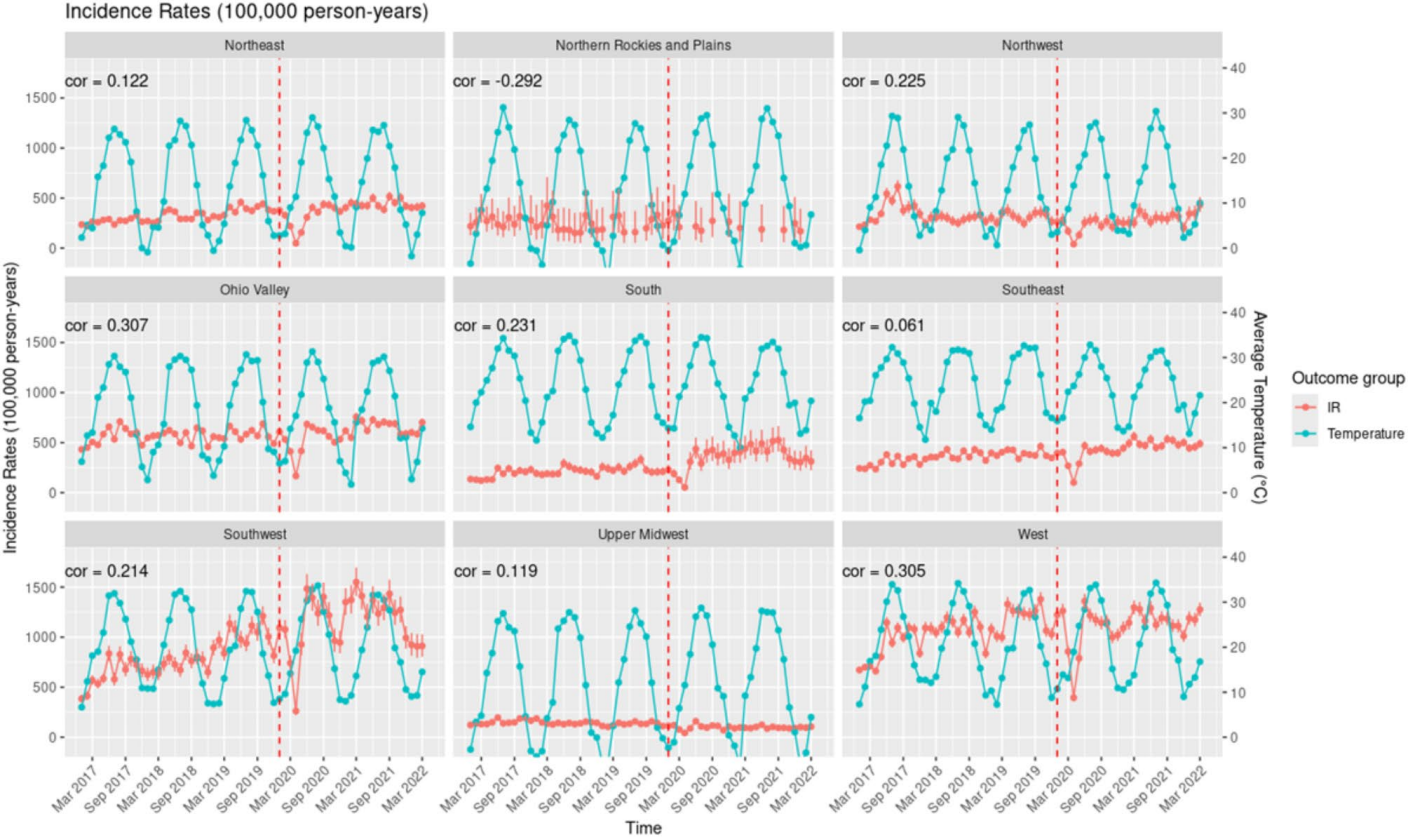
Monthly Incidence Rate of Excess Sun Exposure Stratified by Climate Regions in the United States of America from January 2017 to March 2022.

None of the regions demonstrated a significant correlation between IR of excess sun exposure and fluctuations in maximum regional temperature (Fig. 8). The West (cor = 0.305) displayed a moderate positive correlation, suggesting that increases in regional temperature are moderately associated with higher IR of excess sun exposure. Several regions exhibited weak correlations, including the Southwest (cor = 0.214) and Northwest (cor = 0.218), while the Ohio Valley (cor = 0.168), South (cor = 0.172), Southeast (cor = 0.061), and Upper Midwest (cor = 0.091) reported very weak correlations.

## Discussion

This study analysed trends in the IR of HRI and excess sun exposure among individuals in the U.S. from January 2017 to March 2022 using the PharMetrics Plus for Academics dataset and characterizes individuals with HRI and excess sun exposure. We observe that individuals diagnosed with HRI and excessive sun exposure exhibit distinct demographic profiles and contrasting patterns in the IR of these two conditions.

HRI exhibits a clear seasonal pattern, peaking during the summer months (June–August), consistent with the hottest periods in the US and aligning with previous research^22^. In contrast, excess sun exposure lacks a consistent seasonal trend, with peaks observed in both summer (March, April, June, August, September) and winter (October, November) months. These findings suggest that HRI primarily has environmental drivers, while excess sun exposure could potentially be influenced by behavioural factors over a longer period.

HRI was predominantly diagnosed in younger men, consistent with previous research^1,21,22^. Our estimate of higher male majority (62.3%) closely aligns with Yeargin et al. (2020), who reported 62.7% of HRI patients as male^22^. However, Schmeltz et al. (2016), using the HCUP Nationwide Inpatient Sample (NIS), reported a higher proportion of males (73.6%) hospitalized for HRI compared to our study^21^. This discrepancy may be explained by differences in the study populations resulting from the distinct data sources used. HCUP NIS primarily focuses on inpatient hospitalizations, capturing severe cases of HRI requiring hospitalization, which might disproportionately affect older males with underlying health conditions^40^. PharMetrics Plus for Academics captures commercially insured individuals, capturing a broader spectrum of HRI cases, including milder ones that may not require hospitalization. This could include more women with HRI who are seeking outpatient care or have milder symptoms. Furthermore, our study utilizes a broader range of diagnostic codes (ICD, SNOMED, Read), which may have captured a greater number of female HRI cases.

The HRI cases comprise a significantly larger proportion of individuals aged 0–17 years compared to the excess sun exposure cases. This higher representation of younger individuals is consistent with previous studies and contributes to the lower median age observed in the HRI group^9^.

Despite the summer of 2020 being impacted by COVID-19 restrictions, the highest IR of HRI was observed in the South (comprising Arkansas, Kansas, Louisiana, Mississippi, Oklahoma, and Texas) in August 2020, reaching 186.04 [95% CI: 117.93–279.15]. This high IR in the South during the summer of 2020 is consistent with the fact that 2020 was one of the hottest summers on record in the US, particularly the month of August^41^.

**Fig. 9:**
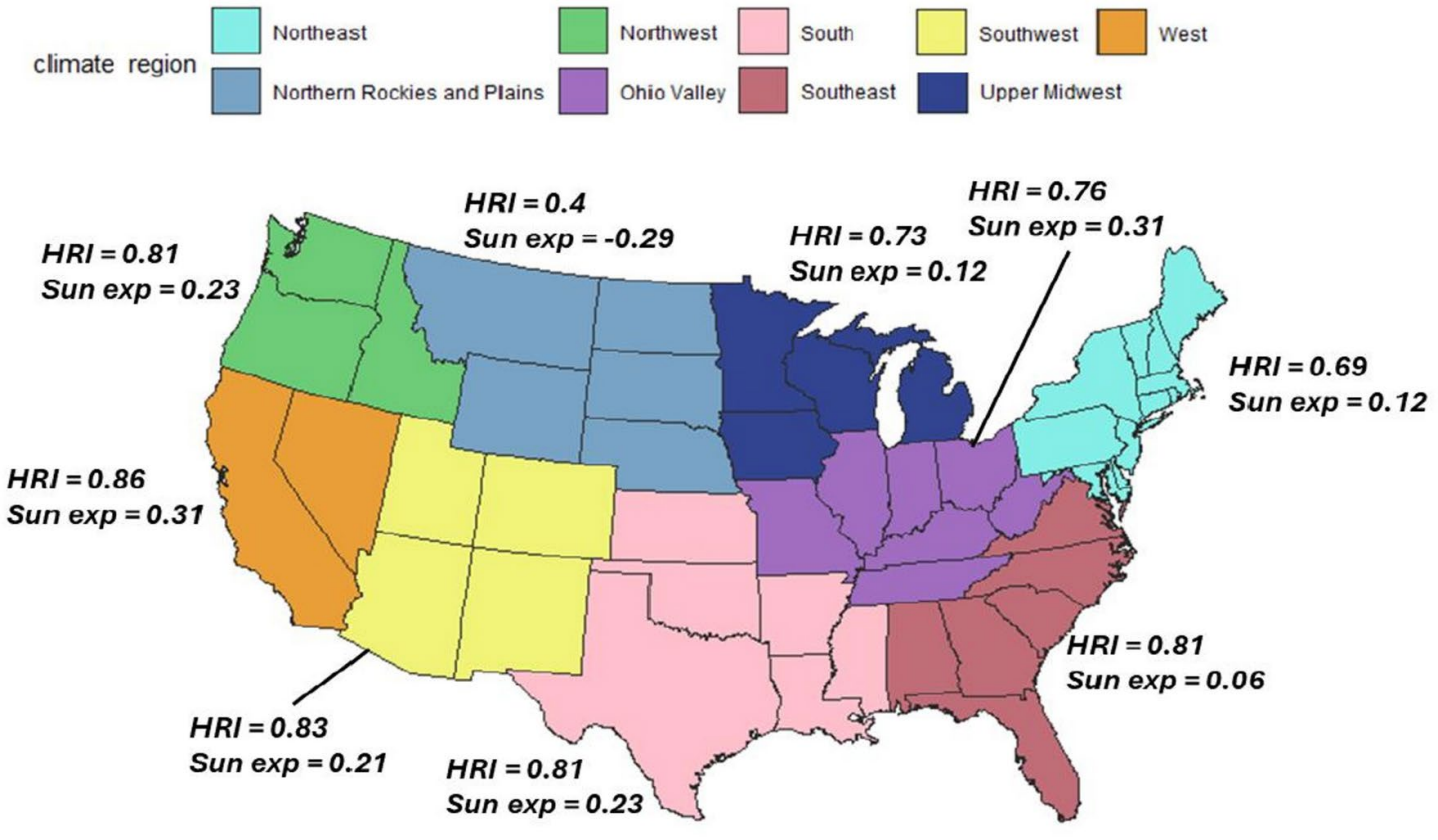
Correlations between Regional Maximum Temperature and the Incidence Rate of Heat Related Illness and Excess Sun Exposure in the Nine Climate Regions of the United States of America.

The West region, encompassing California and Nevada, demonstrated the strongest correlation between regional temperature changes and HRI incidence rates (IR; cor = 0.854), indicating a strong temperature-dependent pattern (Fig. 9). An analysis of heat wave characteristics in fifty large US cities from 1961 to 2023 reveals that in California and Nevada, significant increases in heat wave frequency have been observed, with some locations experiencing 6–8 additional heat waves annually^4^. Concurrently, heat wave intensity in Nevada has risen, with temperature thresholds increasing by 1–1.5°F above historical norms^4^. The heat wave season has also lengthened considerably in California, with many areas experiencing 50–75 additional days of heat wave conditions^4^.

The Southwest, comprising Arizona, Colorado, New Mexico, and Utah, exhibited the second-highest correlation between changes in maximum regional temperature and the IR of HRI. Some areas in Arizona, in particular, exhibit increases of greater than 8 heat waves annually, highlighting a dramatic rise in extreme heat events^4^. Across the Southwest, heat wave duration has increased significantly, with areas in Arizona and New Mexico experiencing 2 to 3 additional days per heat wave event^4^. The heat wave season has extended considerably, with many locations experiencing 50 to 75 additional days of heat wave conditions annually. Analysis of heat wave intensity changes in Phoenix and Salt Lake City reveals significant increases, with temperature thresholds rising by 1.15°F in Phoenix and 1.37°F in Salt Lake City above historical norms^4^.

Extended heat wave seasons, coupled with rising temperatures, drive the strong correlation between regional temperature changes and HRI IR in the West and Southwest. These regions, characterized by unique climates with prolonged droughts, extreme summers, and frequent heat waves, exhibit heightened vulnerability to heat stress. This underscores the critical need for region-specific heat mitigation strategies to address the escalating risk of HRI.

In contrast to HRI, which primarily affects younger males, excess sun exposure was more prevalent among older adults and exhibited a near-equal gender distribution. This suggests that excess sun exposure affects both sexes similarly. The older age profile and higher prevalence of malignant neoplastic diseases in this group are consistent with the cumulative effects of long-term sun exposure, a well-established risk factor for skin cancer development^42,43^.

Interestingly, our analysis revealed no significant correlation between changes in regional temperature and the IR of excess sun exposure. The diagnostic code for excess sun exposure primarily reflects cumulative exposure over time, rather than acute events. Consequently, the excess sun exposure cases majorly consists of elderly individuals who have experienced prolonged sun exposure throughout their lifetime. In contrast, younger individuals are more likely to be classified in the HRI cases where acute heat-related conditions are more prevalent.

The high prevalence of hypertension in both the group suggests its potential role in increasing the groupss’ susceptibility to developing HRI and excess sun exposure. Medications such as beta-blockers and diuretics, commonly used for treating hypertension, may impact the body’s thermoregulation systems, increasing vulnerability to these conditions^25^. The two groups also report a notable uptake of medications used to treat anxiety and depressive disorders which have also been identified as risk factors for HRI previously^1,25^.

Another similarity observed between the HRI and sun exposure groups was the geographic distribution of individuals. The highest proportion of participants in both groups resided in the Ohio Valley region, with 3,105 individuals (29.1%) in the HRI group and 32,260 individuals (24.6%) in the excess sun exposure group. This geographic trend may be linked to long-term climatic shifts observed from 1961 to 2023, particularly changes in heat wave dynamics^4^. The Ohio Valley has experienced a substantial extension in heat wave season length, defined as the number of days between the first and last heat wave of the year, with certain areas reporting over 75 days of heat wave activity annually^4^. The Ohio Valley region has experienced a notable increase in heat wave frequency, with several areas reporting more 6–8 additional heat waves per year compared to historical baselines^4^. Furthermore, heat wave intensity, measured by the increase in temperature above the heat wave threshold (°F), has risen by 1 to 1.5°F in several parts of the region and by more than 1.5°F in others, further contributing to elevated heat exposure risk and potentially influencing the observed health outcomes in this population^4^.

The excess sun exposure group xhibited a significantly larger population size and a greater number of recorded outcomes compared to the HRI group. This disparity suggests that excess sun exposure-related outcomes are more frequently reported, despite potentially encompassing a narrower range of clinical presentations than HRI. Notably, an overlap of 181 individuals was observed between the two groups, indicating shared risk factors in a subset of the population. These shared risks may include environmental factors, such as high regional climate, occupational exposure or underlying comorbidities, which can predispose individuals to both HRI and excess sun exposure-related health issues.

A key strength of this study lies in its use of climate-based regional aggregation. Unlike previous studies that primarily relied on US Census regions or divisions, which may not accurately reflect climatic variations, this study utilizes nine distinct climate regions^22^.

Our study highlights the differential impact of COVID-19 restrictions on HRI and excess sun exposure. Reductions in HRI IR were observed among males aged 18–64 years and females aged 65 years and above, likely driven by decreased outdoor activities and occupational heat exposure. In contrast, while excess sun exposure rates initially declined sharply, they rebounded dramatically to pre-pandemic levels by June 2020, particularly among older adults. This contrasting trajectory suggests that while both outcomes were influenced by pandemic-related disruptions, the underlying mechanisms differ significantly. HRI, primarily driven by environmental factors such as extreme heat and outdoor occupations, demonstrated a more sustained decline. Conversely, excess sun exposure, potentially largely influenced by behavioural factors and given the nature of the outcome taking a longer time to develop, exhibited a rapid recovery, likely reflecting a swift return to pre-pandemic activity levels.

Having identified the key characteristics of these two groups and the differential impact of COVID-19 restrictions on the IR of HRI and excess sun exposure, targeted prevention strategies are warranted. For HRI in general and during future pandemics, interventions should focus on addressing acute risks and prioritize younger, working-age males, particularly those residing in the West and Southwest regions with high heat wave exposure. This necessitates scaling up heat safety protocols, ensuring access to cooling resources, implementing staggered work shifts for outdoor workers, and establishing additional hydration centres. Importantly, these measures should be scaled up during peak summer months when HRI IR is elevated.

Conversely, excess sun exposure prevention efforts should prioritize older adults first while also considering more long-term strategies for younger individuals, as this outcome develops over a longer period. Emphasizing year-round protective behaviours such as consistent sunscreen use, seeking shade during peak sun hours, and minimizing prolonged sun exposure can help reduce the cumulative risks associated with long-term sun exposure.

## Limitations

The PharMetrics Plus for Academics dataset records patient location based on their residence rather than the location where they were diagnosed with HRI or excess sun exposure. This presents a potential limitation, as patients may travel to other states, experience extreme heat or sun exposure events, and subsequently receive a diagnosis outside their state of residence. This discrepancy could impact the accuracy of regional analyses and the interpretation of location-specific risk factors.

In general, this claims dataset includes moderately healthy, employed individuals with access to medical insurance. Since HRI is commonly reported among populations from lower socioeconomic backgrounds who may lack insurance or access to healthcare, the incidence estimates in this study may underestimate the true burden of HRI in the broader population.

The PharMetrics Plus for Academics dataset, mapped to the OMOP-CDM, presents certain limitations. Notably, the absence of sociodemographic data, such as income and occupation, hinders the stratification of the two outcome groups by socioeconomic factors known to influence the incidence of HRI. The lack of detailed residential location information (e.g., city or town, rural vs. urban settings, zip codes) limits the analysis of geographic disparities, given the known association between urban heat islands and HRI risk. Additionally, the exclusion of mortality data precludes the assessment of HRI related mortality within this dataset.

Ethnicity is a crucial factor influencing susceptibility to both HRI and excess sun exposure. Factors such as socioeconomic status, occupational exposures, access to healthcare, cultural practices, and physiological differences like skin pigmentation can significantly vary across different ethnic groups. The absence of ethnicity data in this dataset represents a notable limitation, hindering a comprehensive understanding of the disparities in HRI and excess sun exposure across diverse populations.

Furthermore, the potential misclassification of outcome events in real-world data represents a significant limitation. Not all cases of HRI and excess sun exposure may have been identified or recorded in clinical practice, particularly if patients presented with more severe comorbidities at the time of diagnosis. This may lead to underestimation of the true incidence of these conditions in the dataset.

## Conclusion

Our findings reveal significant differences between the at-risk populations and seasonal patterns reported by HRI and excess sun exposure cases. HRI primarily impacted younger males, particularly those aged 18–64 years, with the South region reporting the highest IR and West and Southwest reporting strong correlation between changes in regional maximum temperature and HRI. In contrast, excess sun exposure was more prevalent among older adults, with a near-equal gender distribution and no clear seasonality nor correlation between changes in regional maximum temperature and the IR of excess sun exposure. Following the COVID-19 pandemic restrictions, the IR of HRI demonstrated a more sustained decline, particularly among younger males, while excess sun exposure rates rebounded sharply following the initial restrictions.

This study is the first to examine HRI using electronic health records correlated with climatic data, while also distinguishing HRI from excess sun exposure. By separating excess sun exposure cases from HRI, we provide a clear and more accurate assessment of HRI, minimizing confounding effects of excess sun exposure cases that may have influenced previous studies. This distinction allows us to identify unique at-risk groups, and seasonal patterns for each outcome.

## Supplementary Information

Supplementary Information 1.

Supplementary Information 2.

Supplementary Information 3.

## Data Availability

All data generated or analysed during this study are included in this published article and its supplementary information files.

## References

[1] Gamboa, L. et al. Analysis of heat stroke and heat exhaustion cases in EudraVigilance pharmacovigilance database. *Eur. J. Clin. Pharmacol.* **79**(5), 679–685. 10.1007/s00228-023-03487-3 (2023). 37009927 10.1007/s00228-023-03487-3 PMC10068193

[2] Thompson, R. et al. Heatwave mortality in Summer 2020 in England: An observational study. *Int. J. Environ. Res. Public Health* **19**(10), 6123 (2022). 35627660 10.3390/ijerph19106123 PMC9141696

[3] Fu, S. H., Gasparrini, A., Rodriguez, P. S. & Jha, P. Mortality attributable to hot and cold ambient temperatures in India: A nationally representative case-crossover study. *PLoS Med.* **15**(7), e1002619. 10.1371/journal.pmed.1002619 (2018). 30040816 10.1371/journal.pmed.1002619 PMC6057641

[4] Climate Change Indicators: Heat Waves. United States Environmental Protection Agency. https://www.epa.gov/climate-indicators/climate-change-indicators-heat-waves#ref11 (Accessed 19 May 2024).

[5] Watts, N. et al. Health and climate change: Policy responses to protect public health. *Lancet* **386**(10006), 1861–1914. 10.1016/s0140-6736(15)60854-6 (2015). 26111439 10.1016/S0140-6736(15)60854-6

[6] N. C. Assessment, The Fifth National Climate Assessment. USA, 2023. https://nca2023.globalchange.gov/ (Accessed on 22 December 2023).

[7] Kingsley, S. L., Eliot, M. N., Gold, J., Vanderslice, R. R. & Wellenius, G. A. Current and projected heat-related morbidity and mortality in Rhode Island. *Environ. Health Perspect.* **124**(4), 460–467. 10.1289/ehp.1408826 (2016). 26251954 10.1289/ehp.1408826 PMC4829994

[8] Schramm, P. J. et al. Heat-related emergency department visits during the northwestern heat wave - United States, June 2021. *MMWR Morb. Mortal. Wkly. Rep.* **70**(29), 1020–1021. 10.15585/mmwr.mm7029e1 (2021). 34292925 10.15585/mmwr.mm7029e1 PMC8297695

[9] Alho, A. M., Oliveira, A. P., Viegas, S. & Nogueira, P. Effect of heatwaves on daily hospital admissions in Portugal, 2000–18: an observational study. *Lancet Planet. Health* **8**(5), e318–e326. 10.1016/s2542-5196(24)00046-9 (2024). 38729671 10.1016/S2542-5196(24)00046-9

[10] Sousa, P. M. et al. Heat-related mortality amplified during the COVID-19 pandemic. *Int. J. Biometeorol.* **66**(3), 457–468. 10.1007/s00484-021-02192-z (2022). 35061075 10.1007/s00484-021-02192-z PMC8780052

[11] Gauer, R. & Meyers, B. K. Heat-related illnesses. *Am. Fam. Phys.* **99**(8), 482–489 (2019). 30990296

[12] Li, T., Horton, R. M. & Kinney, P. L. Projections of seasonal patterns in temperature-related deaths for Manhattan, New York. *Nat. Clim. Change* **3**(8), 717–721 (2013). 10.1038/nclimate1902 PMC4045618 24910717

[13] Sorensen, C. & Hess, J. Treatment and prevention of heat-related illness. *N. Engl. J. Med.* **387**(15), 1404–1413. 10.1056/NEJMcp2210623 (2022). 36170473 10.1056/NEJMcp2210623

[14] Khatana, S. A. M., Werner, R. M. & Groeneveld, P. W. Association of extreme heat with all-cause mortality in the contiguous US, 2008–2017. *JAMA Netw. Open* **5**(5), e2212957–e2212957. 10.1001/jamanetworkopen.2022.12957 (2022). 35587347 10.1001/jamanetworkopen.2022.12957 PMC9121188

[15] Stearns, R. L. et al. Incidence of recurrent exertional heat stroke in a warm-weather road race. *Medicina (Kaunas)* **56**(12), 720. 10.3390/medicina56120720 (2020). 33371206 10.3390/medicina56120720 PMC7766530

[16] Romero, J. J., Clement, P. F. & Beiden, C. Neuropsychological sequelae of heat stroke: Report of three cases and discussion. *Milit. Med.* **165**(6), 500–503. 10.1093/milmed/165.6.500 (2000). 10870374

[17] Taylor, E. V. et al. Differences in heat-related mortality by citizenship status: United States, 2005–2014. *Am. J. Public Health* **108**(S2), S131–S136. 10.2105/ajph.2017.304006 (2018). 29072944 10.2105/AJPH.2017.304006 PMC5920731

[18] Vaidyanathan, A., Malilay, J., Schramm, P. & Saha, S. Heat-related deaths - United States, 2004–2018. *MMWR Morb. Mortal. Wkly. Rep.* **69**(24), 729–734. 10.15585/mmwr.mm6924a1 (2020). 32555133 10.15585/mmwr.mm6924a1 PMC7302478

[19] O’Neill, M. S., Zanobetti, A. & Schwartz, J. Disparities by race in heat-related mortality in four US cities: The role of air conditioning prevalence. *J. Urban Health* **82**, 191–197 (2005). 15888640 10.1093/jurban/jti043 PMC3456567

[20] Sera, F. et al. Air conditioning and heat-related mortality: A multi-country longitudinal study. *Epidemiology* **31**(6), 779–787 (2020). 33003149 10.1097/EDE.0000000000001241

[21] Schmeltz, M. T., Petkova, E. P. & Gamble, J. L. Economic burden of hospitalizations for heat-related illnesses in the United States, 2001–2010. *Int. J. Environ. Res. Public Health* **13**(9), 894 (2016). 27618079 10.3390/ijerph13090894 PMC5036727

[22] Yeargin, S., Hirschhorn, R. & Grundstein, A. Heat-related illnesses transported by United States emergency medical services. *Medicina* **56**(10), 543 (2020). 33080867 10.3390/medicina56100543 PMC7602997

[23] Kim, S., Kim, D. H., Lee, H. H. & Lee, J. Y. Frequency of firefighters’ heat-related illness and its association with removing personal protective equipment and working hours. *Ind. Health* **57**(3), 370–380. 10.2486/indhealth.2018-0063 (2019). 30210098 10.2486/indhealth.2018-0063 PMC6546580

[24] Heinzerling, A. et al. Risk factors for occupational heat-related illness among California workers, 2000–2017. *Am. J. Ind. Med.* **63**(12), 1145–1154. 10.1002/ajim.23191 (2020). 33075156 10.1002/ajim.23191

[25] Layton, J. B. et al. Heatwaves, medications, and heat-related hospitalization in older Medicare beneficiaries with chronic conditions. *PLoS One* **15**(12), e0243665. 10.1371/journal.pone.0243665 (2020). 33301532 10.1371/journal.pone.0243665 PMC7728169

[26] Kalisch Ellett, L. M., Pratt, N. L., Le Blanc, V. T., Westaway, K. & Roughead, E. E. Increased risk of hospital admission for dehydration or heat-related illness after initiation of medicines: A sequence symmetry analysis. *J. Clin. Pharm. Ther.* **41**(5), 503–507. 10.1111/jcpt.12418 (2016). 27378245 10.1111/jcpt.12418

[27] Martin-Latry, K. et al. Psychotropic drugs use and risk of heat-related hospitalisation. *Eur. Psychiatry* **22**(6), 335–338. 10.1016/j.eurpsy.2007.03.007 (2007). 17513091 10.1016/j.eurpsy.2007.03.007

[28] Wilhelmi, O. V., Howe, P. D., Hayden, M. H. & O’Lenick, C. R. Compounding hazards and intersecting vulnerabilities: Experiences and responses to extreme heat during COVID-19. *Environ. Res. Lett.* **16**(8), 084060. 10.1088/1748-9326/ac1760 (2021).

[29] Faurie, C., Varghese, B. M., Liu, J. & Bi, P. Association between high temperature and heatwaves with heat-related illnesses: A systematic review and meta-analysis. *Sci. Total Environ.* **852**, 158332. 10.1016/j.scitotenv.2022.158332 (2022). 36041616 10.1016/j.scitotenv.2022.158332

[30] Radgoudarzi, N. et al. Medical, environmental, and social determinants associated with periocular cutaneous malignancies in the united states using the all of us national database. *Cureus* **16**(7), e65831. 10.7759/cureus.65831 (2024). 39219888 10.7759/cureus.65831 PMC11363474

[31] Ivry, G. B., Ogle, C. A. & Shim, E. K. Role of sun exposure in melanoma. *Dermatol. Surg.* **32**(4), 481–492 (2006). 16681655 10.1111/j.1524-4725.2006.32101.x

[32] Oliveria, S. A., Saraiya, M., Geller, A. C., Heneghan, M. K. & Jorgensen, C. Sun exposure and risk of melanoma. *Arch. Dis. Childhood* **91**(2), 131–138. 10.1136/adc.2005.086918 (2006). 16326797 10.1136/adc.2005.086918 PMC2082713

[33] IQVIA. IQVIA PharMetrics® Plus for Academics Enhanced with Mortality Data. https://www.iqvia.com/locations/united-states/library/fact-sheets/iqvia-pharmetrics-plus-for-academics-enhanced-with-mortality-data (Accessed 01 November, 2024).

[34] Ma, Q. et al. Patterns of use of systemic therapies among patients with metastatic melanoma: A retrospective claims database analysis in the United States. *J. Dermatol. Treat.* **28**(6), 549–553. 10.1080/09546634.2016.1277176 (2017). 10.1080/09546634.2016.1277176 28100090

[35] Hallinan, C. M. et al. Seamless EMR data access: Integrated governance, digital health and the OMOP-CDM. *BMJ Health Care Inform.*10.1136/bmjhci-2023-100953 (2024). 38387992 10.1136/bmjhci-2023-100953 PMC10882353

[36] Biedermann, P. et al. Standardizing registry data to the OMOP Common Data Model: Experience from three pulmonary hypertension databases. *BMC Med. Res. Methodol.* **21**, 1–16 (2021). 34727871 10.1186/s12874-021-01434-3 PMC8565035

[37] N. C. f. E. Information. Climate at a Glance Regional Time Series. NOAA’s National Centers for Environmental Information (NCEI). https://www.ncei.noaa.gov/access/monitoring/climate-at-a-glance/regional/time-series/105/tavg/12/0/2017-2022?base_prd=true&begbaseyear=2015&endbaseyear=2024 (Accessed 3, November 2024).

[38] US Climate Regions. National Centers for Environmental Information. https://www.ncei.noaa.gov/access/monitoring/reference-maps/us-climate-regions (Accessed 30 October, 2024).

[39] T. Karl and W. J. Koss, Regional and national monthly, seasonal, and annual temperature weighted by area. 1895–1983 (1984).

[40] A. f. H. R. a. Quality. NIS Overview. Agency for Healthcare Research and Quality. https://hcup-us.ahrq.gov/nisoverview.jsp (Accessed 29 November, 2024).

[41] N. O. a. A. Administration. Summer 2020 ranked as one of the hottest on record for U.S. August was remarkably hot and destructive. https://www.noaa.gov/news/summer-2020-ranked-as-one-of-hottest-on-record-for-us#:~:text=August%202020%20will%20be%20remembered,derecho%20and%20raging%20wildfires%20out (Accessed November 21, 2024).

[42] Narayanan, D. L., Saladi, R. N. & Fox, J. L. Ultraviolet radiation and skin cancer. *Int. J. Dermatol.* **49**(9), 978–986. 10.1111/j.1365-4632.2010.04474.x (2010). 20883261 10.1111/j.1365-4632.2010.04474.x

[43] Raimondi, S., Suppa, M. & Gandini, S. Melanoma epidemiology and sun exposure. *Acta Derm. Venereol.* **100**(11), adv00136. 10.2340/00015555-3491 (2020). 32346751 10.2340/00015555-3491 PMC9189754

